# Genome-Wide Association Study on the Estimated Breeding Values for Udder and Longevity and the Candidate Genes in Holstein-Friesian Cows in Hungary

**DOI:** 10.3390/ani16010073

**Published:** 2025-12-26

**Authors:** Attila Zsolnai, László Bognár, Szabolcs Albin Bene, Laszló Rózsa, Péter Póti, Ferenc Szabó, István Anton

**Affiliations:** 1 Department of Animal Breeding, Institute of Animal Sciences, Hungarian University of Agriculture and Life Sciences, Guba Sándor utca 40, H-7400 Kaposvár, Hungary; 2 National Association of Hungarian Holstein Friesian Breeders, Lőportár utca 16, H-1134 Budapest, Hungary; bognar@holstein.hu; 3 Department of Animal Breeding, Institute of Animal Sciences, Hungarian University of Agriculture and Life Sciences, Deák Ferenc utca 16, H-8360 Keszthely, Hungary; bene.szabolcs.albin@uni-mate.hu (S.A.B.); rozsa.laszlo@uni-mate.hu (L.R.); anton.istvan@uni-mate.hu (I.A.); 4 Department of Animal Husbandry Technology and Animal Welfare, Institute of Animal Sciences, Hungarian University of Agriculture and Life Sciences, Páter Károly utca 1, H-2100 Gödöllő, Hungary; poti.peter@uni-mate.hu; 5 Albert Kázmér Faculty of Mosonmagyaróvár, Széchenyi István University, Vár tér 2, H-9200 Mosonmagyaróvár, Hungary; szabo.ferenc@sze.hu

**Keywords:** bovine, genomic selection, genetic marker, mammary gland, productive lifespan, genetic potential

## Abstract

Our genome-wide association study identified single-nucleotide polymorphisms (SNPs) associated with estimated breeding values (EBVs) for udder traits and longevity in Holstein-Friesian cows. While no SNP was individually associated with multiple EBVs, the functional profiles of the associated genes revealed overlapping biological processes across traits, including cell signaling, transcription regulation, immune response, metabolism, and cellular maintenance. Notably, nearby SNPs BTB-01738708 and ARS-BFGL-NGS-111478 were associated with EBV_longevity_ and EBV_udder_ and located near numerous genes, including *GPR85*, *BMT2*, *IFRD1*, and *DOCK4,* suggesting a potential for shared genetic influence on these traits. Our findings provide insights into the complex genetic architecture of these economically important traits and highlight the need for further research, including fine-mapping and functional genomics, to elucidate the specific variants and their effects.

## Simple Summary

We analyzed the genome of Hungarian Holstein-Friesian cows to identify genomic regions that affect the udder and longevity. None of the investigated single-nucleotide polymorphisms were associated with more than one of the udder and longevity traits in the sampled animals. There was one region that had overlapping genes within one million base pairs of two single-nucleotide polymorphisms associated with udder or longevity.

## 1. Introduction

Holstein-Friesian (HF) cows are recognized for their excellent milk production and adaptability to diverse environmental conditions [1]. The breeding activity of HF cattle in Hungary is promoted and coordinated by the National Association of Hungarian Holstein Friesian Breeders (NAHHFB), which was established in 1989 [2]. The HUNGENOM project was introduced by NAHHFB in 2019, to supports genomic selection through genomically enhanced breeding value estimation [2].

### 1.1. Studies Related to Udder Health and Conformation

Among several traits [3] udder health is of crucial importance for dairy cattle productivity and longevity. Teat shape, such as triangular barrel shape and pointed teat end, has been associated with an increased risk of clinical mastitis [4]. The *PEPD* and *ZFC3H1* genes were identified as genetic markers related to tilted udder floor in Ayrshire cattle [5]. Four quantitative trait loci (QTLs) in Gir dairy cattle were demonstrated to affect udder conformation traits; two were associated with udder width (BTA14 and BTA20), and two were associated with udder depth (BTA2 and BTA18) [6]. Single-nucleotide polymorphisms (SNPs) rs454303072, rs382671389, and rs435289107 have been associated with udder conformation traits in Sahiwal (*Bos indicus*) and Karan Fries (*Bos taurus* × *Bos indicus*) cattle in India [7]. Numerous genes, including *MMS22L*, *E2F8*, *CSRP3*, *CDH11*, *PEX26*, *HAL*, *TAMM41*, *HIVEP3*, *SBF2*, *MYO16*, and *STXBP6,* have been identified as candidate genes for teat conformation traits in Chinese HF cows [8]. Several QTLs in Montbéliarde, Normande, and Holstein breeds were significantly associated with udder traits. These candidate genes were located on BTA5 (*ABCC9*), BTA6 (*GC*), and BTA14 (*PLAG1*) [9]. Another study in the same dairy cattle breeds in France identified 10 possible candidate genes for udder conformation traits (*ESR1*, *FGF2*, *FGFR2*, *GLI2*, *IQGAP3*, *PGR*, *PRLR*, *RREB1*, *BTRC*, and *TGFBR2*) [10]. Fifteen SNPs on BTA5 were associated with udder support scores in Nellore–Angus crossbred cows. Some were located within genes (*VDR*, *PTPRR,* and *IL22*), whereas others were in the vicinity of known genes (*SPCS3* and *DYRK2*) [11].

### 1.2. Studies Related to Longevity

Studies on the relationship between longevity and fertility traits in HF cows in China, have proposed four fertility traits (days open, interval from calving to first insemination, interval from first to last inseminations, and calving ease) as indirect indicators of longevity traits [12]. Concerning the association between the age at first calving and longevity of HF cows in Japan, it has been concluded that younger cows at first calving (<22.5 months) had better survivability and higher overall lifetime milk yield than older cows (≥25.5 months) [13]. The g.9422T>C variant in the *TLR4* gene (rs8193060) in Czech Simmental cattle was found to be associated with four reproductive traits, including production longevity [14]. Given the short longevity of the Shanghai Holstein cattle population, two methods (integrated haplotype score and runs of homozygosity) have been used to identify genomic selection signatures. Several candidate genes have been identified (e.g., *IL22RA1*, *CALHM3*, *ITGA9*, *NDUFB3*, *RGS3*, *SOD2*, *SNRPA1*, *ST3GAL4*, *ALAD*, *EXOSC10*, and *MASP2*), which proved to be associated with adaptation and economic traits [15].

Given the complexity of longevity traits in Chinese HF cattle, a GWAS investigated both the full lifespan traits and the partial productive life trait. The study identified numerous candidate genes for longevity, including *RPRM*, *GRIA3*, *GTF2H5*, *CA5A*, *CACNA2D1*, *FGF10*, and *DNAJA3* [16]. A GWAS performed in the HF cattle in Italy revealed two QTLs on BTA16 and BTA30 containing 10 candidate genes associated with three indicators of longevity (days in production, days in herd, number of calvings) [17]. A GWAS performed in North American Holstein cattle for lifetime profit index, lactation persistency, and longevity highlighted some previously proposed candidate genes (*DGAT1*, *GRINA*, and *CPSF1*), as well as new ones, including *SLC2A4RG* and *THRB* [18]. A single-marker GWAS revealed five loci highly associated with longevity in a composite beef cattle breed (50% Red Angus, 25% Charolais, and 25% Tarentaise): ARS-BFGL-BAC-15059 on BTA1, ARS-BFGL-NGS-104159 on BTA3, ARS-BFGL-NGS-32882 on BTA9, UA-IFASA-7571 on BTA19, and ARS-BFGL-NGS-32883 on BTA25 [19].

A whole-genome sequencing study in dairy cattle breeds, identified seven genomic regions in HF cattle and five in Red Dairy cattle associated with longevity. One genomic region on BTA6 overlapped with *NPFFR2* (a candidate gene for mastitis), whereas another region on BTA18 overlapped with *ZNF717* and *ZNF613* (associated with calving difficulties) [20]. Nine economically important traits (including longevity and milk production traits) have been investigated in HF cattle in Italy. Several genes were found to be associated with the examined traits (e.g., *PRLR*, *ACACA*, *CRH*, *CXCR1*, *FASN*, *GH1*, *LEP*, *LGB*, *MFGE8*, *SRC*, *TG*, *THRSP*, and *TPH1*) [21]. Nine functional SNPs located within five different genes have been examined for their possible association with functional longevity. The results showed a strong association between the *LEP* gene and longevity in Polish HF cows [22]. A significant effect of the *CAST* gene on the fertility and longevity of dairy cattle has also been described [23].

Identification of genomic regions that affect the udder and longevity in dairy cattle represents a valuable tool for enhancing breeding strategies and genetic selection. Regarding the importance of udder and longevity in the dairy industry, this study investigated the association of SNPs on EBVs for udder and longevity traits in Hungarian HF cows.

## 2. Materials and Methods

This study did not require approval from the Ethical Committee on Animal Experiments since its data were acquired through genotyping that is the routine breeding procedure coordinated by the NAHHFB.

All phenotypic and genotypic data were supplied by the NAHHFB. Genotyping of Hungarian HF cows was performed with the EuroG_MDv4 microarray (Eurogenomics, Amsterdam, The Netherlands), containing 67,227 SNPs. Following quality control, we filtered out both samples and SNPs that exhibited a call rate lower than 0.95 and minor allele frequency below 0.05. The final dataset consisted of 2963 individuals and 87.99% (59,151) of the total SNPs.

The examined trait udder was defined according to the current (June 2023) standard trait definition for dairy cattle [24] of the International Committee for Animal Recording (ICAR). The standard traits for evaluating the udder are fore udder attachment, rear udder height, central ligament, udder depth, texture, rear udder width, front teat placement, teat length, and rear teat placement. From these traits indexes are formed. The EBV for udder was calculated as described by Bognar et al. [25]. The EBV was based on the index and EBV was used in the GWAS.

The following linear random regression model is used to directly calculate the EBV for longevity, where survival per month is analyzed:

*Y_ijklmno_* = *HYS*_*LS_i_* + *YSAM*_*LS_j_* + *HSC_k_* + *het_l_* + *rec_m_* + *animal_n_* + *rest_ijklmno_* (1)

where *Y_ijklmno_* is the observation for survival in month *o* after first calving [mo = 1–72]; *HYS*_*LS*_*i*_ is the herd-year-season × lactation-stage *i* (year-season of first calving, lactation divided into 1, 2, and ≥3+, and stage of lactation divided into months 1–2, 3–9, ≥10+, and the dry period).

*YSAM*_*LSj* is the year-season × age of first calving × within-herd production level × lactation-stage *j* (year-season of first calving; age at first calving in months 21, 22, …, 34, ≥35; the within-herd production level is defined per three years and is divided into five classes [20% each] for predicted or realized age-corrected 305-day yield of kg fat and protein).

*HSC*_*k*_ is the herd size change *k*, which is calculated by comparing the number of cows present in a herd in year *y* with the number of cows in the same herd in year *y* + 1 (seven classes were defined: shrinkage between 90% and 50%, shrinkage between 50% and 30%, shrinkage between 30% and 10%, neither shrinkage nor growth greater than 10%, growth between 10% and 30%, growth greater than 30%, and herds that were terminated [>90% shrinkage]).

*het_l_* is heterosis *l* of animal *n*.

*rec_m_* is recombination *m* of animal *n*, which captures the impact of an animal’s number of parental recombination events.

*animal*_*n*_ is the additive genetic effect (or breeding value) of animal *n*, which is estimated using a random regression function and describes the effect on survival for every month between 1 and 72; *rest*_*ijklmno*_ is the residual of *Y*_*ijklmno*_ (i.e., anything that is not explained by the model).

In the GWAS, animals were sorted based on their EBVs for udder and longevity (EBV_udder_, and EBV_longevity_, respectively). Initially, each EBV category was split into high and low tail groups. The EBV_udder_ included 5%, EBV_longevity_ 7% of the genotyped individuals in both of their corresponding tail groups. The genetic distance values between the high and low tail groups were 0.007 and 0.005 for the categories of udder and longevity, respectively. Means and standard deviations for EBVs of udder and longevity are 0.638 ± 0.558 and 0.330 ± 0.539. The cutoff values for inclusion in a tail were: EBV_udder_high_ > 1.25, EBV_uder_low_ < 0.03; EBV_longevity_high_ > 0.99, EBV_longevity_low_ < −0.38. For each trait (EBV_udder_, and EBV_longevity_ we used SNP and Variation Suite (SVS) software (version 8.8.1; Golden Helix, Bozeman, MT, USA) to compute genetic distance of high and low tail groups, genetic distance of SNPs [F_st_marker_], linear regression [26], and haplotype association tests [27]).

**Figure 1:**
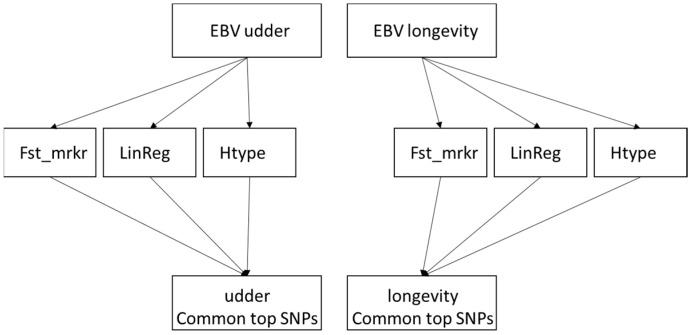
The graphic illustrates the methodologies for identifying single-nucleotide polymorphisms (SNPs) connected to estimated breeding values (EBVs) for udder and longevity. A variety of analytical approaches were employed for each EBVs, such as linear regression (LinReg), haplotype association (Htype), and the measurement of genetic distance between SNPs (Fst_mrkr). In the final step, the most impactful SNPs for each trait were determined by consolidating common hits from these tests.

The procedure is presented as a flow diagram in Figure 1.

**Figure 2:**
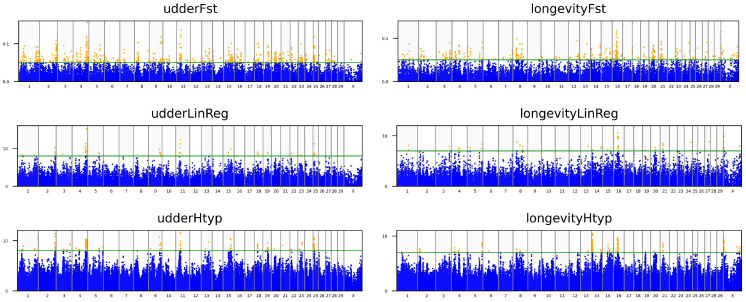
Manhattan plots of trait-test results. The layout is organized by both trait and analytical method: Columns represent the traits: udder (EBV_udder_) on the left and longevity (EBV_long_) on the right. Rows represent the methods: The top row shows F_st_marker_ results, the middle row shows linear regression, and the bottom row shows haplotype associations. In each plot, the green line indicates the threshold. Any SNPs positioned above this line (orange dots) are kept to identify common hits.

For the haplotype association analysis, chi-squared test was applied to each haplotype within a predefined five-marker window [28]. The construction of these haplotypes was accomplished using the expectation-maximization algorithm, which was set to run for a maximum of 50 iterations with a convergence tolerance of 0.0001. After checking for the criteria to have the false discovery rate of the SNPs below 0.02, for the udder (EBV_udder_) and longevity (EBV_longevity_) associations, thresholds were established. The threshold values for F_st_marker_, the −log_10_(*p*) from the linear regression and from haplotype tests were defined as EBV_udder_: 0.05, 8, and 8; EBV_longevity_: 0.05, 7, and 7 (Figure 2). Single-nucleotide polymorphisms that exceeded these values were then identified as significant. This procedure pinpointed a total of 62 SNPs. The false discovery rates for these SNPs were far below 0.02, ranging from 1.9 × 10^−21^ to 1.0 × 10^−4^. We investigated genes within a 1 Mb range, upstream and downstream, of the detected SNPs, referencing the ARS-UCD1.2 B. taurus genome assembly (Supplementary Table S1).

## 3. Results

Thirty-four SNPs associated with EBV_udder_ were identified on BTAs 1, 2, 4, 5, 9, 11, 15, 18, 19, 22, and 25. Twenty-eight SNPs associated with EBV_longevity_ were identified on BTAs 4, 5, 8, 13, 14, 16, 19, 21, and X (Table 1). For the result grouped by the order of the chromosomes and positions, see Supplementary Tables S1–S3. Both Supplementary Tables S2 and S3 carry regression beta, the effect size on a particular trait revealing both the strength and direction of the association between a single-nucleotide polymorphism and the phenotype of interest.

The maximum values of the identified SNPs were 0.136 for *F*_st_marker_, 12.31 for the −log_10_(*p*) of the linear regression, and 11.6 for the −log_10_(*p*) of the haplotype association tests on BTA 11, in case of EBV_udder_. In case of EBV_longevity_ these values were 0.118, 10.6 on BTA 16 and 10.2 on BTA 14, respectively.

Full names of the genes are listed in Supplementary Table S1. Values of F_st-marker_, −log_10_(*p*) derived linear or haplotype regression, regression beta, and false discovery rates are presented in Supplementary Table S2, while the frequencies of the alleles can be tracked in Supplementary Table S3. Tables S4–S6 contain the closest genes to the reported SNPs (Supplementary Table S1) and some of their properties.

Table 2 summarizes the studied traits and classifies the associated genes by function, highlighting those reported involved in multiple functions.

Functional categories found in the literature are summarized in Table 2. Genes falling into tissue structure, immune response, metabolism, responses to internal and external stimuli, regulation of gene expression and differentiation, and signaling categories were identified both in cases of EBV_udder_ and EBV_longevity_. Two categories, transport and signalling were presented only in case of EBV_udder_, while EBV_longevity_ harboured additional genes belonging to cellular health and genomic integrity, reproductive success, and responding to environmental and physiological stress categories.

## 4. Discussion

The objective of this research was to pinpoint genome-wide suggestive SNPs linked to EBVs for udder and longevity traits. We employed a trio of algorithms (Figure 1) to locate these associations, then used the most significant results (Figure 2) to determine the candidate genes. None of the 62 identified SNPs were associated with both EBVs, suggesting that different sets of genes influence each trait. Two SNPs were located within 0.965 million base pairs of each other, with one associated with EBV_longevity_ (BTB-01738708) and the other associated with EBV_udder_ (ARS-BFGL-NGS-111478). However 0.965 Mb is over the reported, average 50 to 100 kilobase distance, which refers to the 0.2–0.3 average r^2^ value in Holstein [29], and in general the linkage is decaying exponentially by distance, there are long range linkage disequilibrium regions typically covering 1–5 Mb between haplotype blocks [30] reported in French cattle breeds.

**Figure 3:**
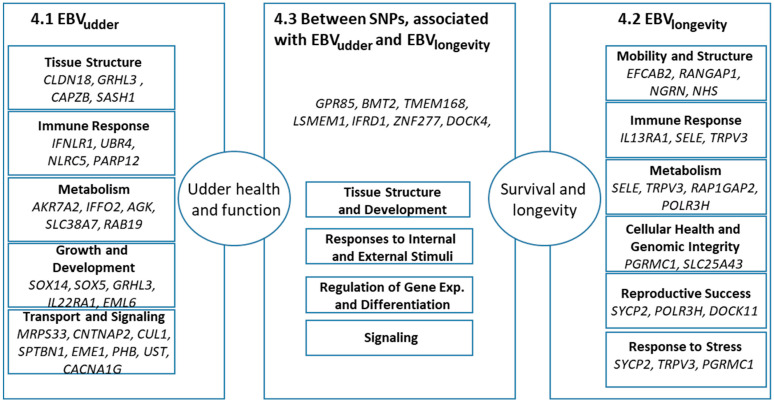
Summary of the candidate genes close to SNPs associated with EBV_udder_ and EBV_longevity_ in HF and their reported processes. The left and right panels detail the specific genes and functional categories linked to each trait, converging on ‘Udder health and function’ and ‘Survival and longevity’. The central panel highlights genes located between two SNPs. Numbers in the panel titles reflect the numbering of the headings of the text below.

The visual representation of the following text is Figure 3. It serves as a guide for the reader’s understanding of the structural organization of the findings found in the literature related to surrounding genes of the reported SNPs.

### 4.1. Genes Around SNPs Associated with EBV_udder_

#### 4.1.1. Tissue Structure

Proteins such as CLDN18 are involved in tight junctions, which are crucial for maintaining the barrier function of epithelial tissues [31]. The mammary gland epithelium forms a critical barrier against pathogens, and it is essential for preventing mastitis. GRHL3 also plays a role in epithelial development and barrier formation [32]. CAPZB is involved in organizing the actin cytoskeleton, which is important for cell shape, movement, and tissue structure [33]. SASH1 has been associated with cell adhesion and cytoskeletal organization [34].

#### 4.1.2. Immune Response

Several genes were implicated in the immune responses within the udder. IFNLR1 is part of the receptor system for type III interferons, which play a significant role in innate immunity at epithelial surfaces [35], including potentially in the mammary gland’s defense against infection. UBR4 is involved in immune responses, as seen in its association with brucellosis resistance [36]. NLRC5 plays a role in innate immunity and has anti-inflammatory effects [37]. PARP12 also exhibits antiviral activity [38], which could be relevant to udder health.

#### 4.1.3. Metabolism

Milk production is a metabolically demanding process requiring efficient nutrient uptake and utilization [39]. AKR7A2 is associated with oxidative stress [40], which impacts metabolic processes [41]. *IFFO2* is a candidate gene for residual body weight gain [42], suggesting a link to overall metabolism. AGK is involved in lipid metabolism [43]. SLC38A7 is an amino acid transporter [44], pointing to its role in milk synthesis [45]. RAB19 correlates positively with milk fat globule size [46], indicating a role in lipid secretion.

#### 4.1.4. Growth, Development, and Differentiation

The development of the mammary gland is a highly regulated process involving cell growth and differentiation. SOX14 and SOX5 (SRY-box transcription factors) are members of the SOX gene family known for their roles in development and cell differentiation [47,48]. GRHL3 also contributes to embryonic development [49]. IL22RA1 is linked to growth traits [15,50], which is relevant to mammary gland development. EML6 is involved in oocyte meiotic division [51], highlighting a connection to reproductive processes that are intrinsically linked to lactation.

#### 4.1.5. Transport and Signaling

Proper udder function relies on the efficient operation of cellular processes [52]. MRPS33 is a mitochondrial protein crucial for cellular energy production [53]. CNTNAP2 is suspected of being involved in gastrointestinal function and neurological processes [54], potentially indicating broader cellular communication roles. CUL1 is part of a protein ubiquitination complex involved in protein degradation and regulation of various cellular processes [55]. SPTBN1 is a cytoskeletal protein with diverse functions, including cell signaling [56]. EME1 is involved in DNA repair [57], which is essential for maintaining genomic stability in actively dividing cells. PHB has been associated with cell proliferation and signaling [58]. UST is involved in synthesizing glycosaminoglycans [59], which are components of the extracellular matrix and play roles in cell signaling and maintenance of tissue structure [60]. CACNA1G is a calcium channel component [61], and calcium signaling is vital for many cellular processes, including milk secretion.

### 4.2. Genes Around SNPs Associated with EBV_longevity_

While the listed proteins associated with cattle longevity exhibit diverse functions across different species and biological contexts, they are all involved in fundamental biological processes that jointly shape an animal’s ability to survive, remain healthy, reproduce, and be productive over an extended period [62].

#### 4.2.1. Mobility and Structural Soundness

The ability to move freely and bear weight is crucial for grazing, accessing feed, and overall well-being [63]. Proteins like EFCAB2 and RANGAP1, involved in bone development [64] and differentiation [65], contribute to skeletal health. NGRN, associated with a neuromuscular disorder [66], directly impacts mobility. While NHS has been associated with the development of head organs [66], it may be crucial for proper development, which in turn influences overall health and function. Sound feet, legs, and a robust skeletal system reduce the risk of lameness, a significant reason for culling [67].

#### 4.2.2. Immune Response

The ability to fight off infections is vital for longevity [68]. IL13RA1 is involved in the response to bacterial infection [69]. SELE’s role in inflammation and immune cell recruitment [70] is also critical for disease defense. A robust immune system helps cattle resist common diseases [71].

#### 4.2.3. Metabolism

Several genes are associated with metabolic processes that influence body condition, energy utilization, and resilience. SELE and TRPV3 are involved in inflammation [70] and lipolysis [72], processes critical for energy mobilization and metabolic responses. RAP1GAP2 is associated with obesity-related traits [73], further emphasizing the link between metabolism and long-term health. POLR3H has been associated with fatty acid composition [74]. Efficient and balanced metabolism is essential for sustained high productivity and overall health [75], contributing to a longer productive life.

#### 4.2.4. Cellular Health and Genomic Integrity

PGRMC1 and SLC25A43 have been associated with resistance to environmental and oxidative stress [76] and identified as a target of oxidative toxicity [77], respectively, highlighting the importance of cellular defense mechanisms against damaging agents that accumulate over time.

#### 4.2.5. Reproductive Success

Reproductive efficiency is a major determinant of longevity in a production setting [78]. Proteins like SYCP2, which is involved in epididymal function and sperm quality [79]; POLR3H, which influences ovarian function [80] and fertility [74]; and *DOCK11,* which correlates with days open [81], directly impact reproductive performance. Cows that rebreed successfully and calve regularly are more likely to remain in the herd [82].

#### 4.2.6. Responding to Environmental and Physiological Stress

Cattle are exposed to various stressors throughout their lives. SYCP2 appears to be involved in the heat stress response [83]. TRPV3 is associated with thermoception [84] and metabolic processes [72], potentially influencing how animals cope with temperature fluctuations. PGRMC1’s role in environmental and oxidative stress resistance [76] is directly related to an animal’s ability to withstand challenging conditions. The capacity to manage stress effectively contributes to better health and longevity [85].

### 4.3. Genes Between SNPs Associated with EBV_udder_ and EBV_longevity_

#### 4.3.1. Tissue Structure and Development

IFRD1 is involved in urothelial cell regeneration, skeletal muscle regeneration, and bone homeostasis and has been associated with a congenital anomaly (craniosynostosis) and growth [86,87,88,89,90,91]. EFCAB2 (mentioned in Section 4.3.1 but also relevant in development) enhances osteoblast differentiation and bone mineralization. DOCK4 influences goblet cell differentiation in the intestine [92].

#### 4.3.2. Responses to Internal and External Stimuli

Proteins are often involved in how cells and organisms react to their environment or internal state [93]. BMT2 senses S-adenosylmethionine, a metabolite derived from the amino acid methionine, reflecting nutrient availability [94]. *GPR85* expression is influenced by obesity [95], suggesting a link to metabolic state. TMEM168 is involved in the response to methamphetamine [96]. The expression of *LSMEM1* is altered by bisphenols [97]. IFRD1 is a developmental regulator but is also linked to inflammatory responses and regeneration [87,88].

#### 4.3.3. Regulation of Gene Expression and Differentiation

Proteins like IFRD1 are explicitly linked to regulating developmental processes, differentiation [98], and gene expression, acting as a transcriptional co-regulator [99]. MiRNAs, which regulate gene expression and target genes like *GPR85* [100] and *IFRD1* [87], highlight another layer of regulation. ZNF277, a zinc finger protein, likely functions as a transcription factor [101], directly influencing gene expression. The expression of *LSMEM1* is influenced by environmental chemicals [97], suggesting its involvement in cellular responses and regulation.

#### 4.3.4. Signaling

GPR85 is a G protein-coupled receptor, a major class of proteins involved in transmitting signals from outside the cell to the inside [102]. BMT2 functions as a sensor for S-adenosylmethionine, influencing MTORC1 signaling and thereby regulating cell growth and metabolism [94]. DOCK4 is involved in coordinating secretion and regulating intercellular connections [92,103], both of which are critical aspects of cell communication and tissue organization. TMEM168’s effect on GABA levels [96] also points to a role in neuronal signaling.

### 4.4. Impressions Based on Recurring Functional Categories Across the Two EBVs

Genes encoding proteins involved in cell signaling (e.g., *IL22RA1*, *SASH1*, and *DOCK11*), transcription regulation (e.g., *SOX14*, *GRHL3*, and *POLR3H*), immune response (e.g., *IFNLR1*, *IL22RA1*, *UBR4*, *NLRC5*, *SELE*, *DOCK11*, and *IL13RA1*), metabolism (e.g., *AKR7A2*, *AGK*, *SLC38A7*, *PRELID3B*, *TRPV3*, and *PGRMC1*), and cellular maintenance and repair (e.g., *CAPZB*, *IMMP2L*, *PARP12*, *KDM7A*, *EME1*, *NGRN*, *RANGAP1*, and *SLC25A43*) appear to be associated with the investigated traits.

Our findings confirms previously reported association of the *DOCK11* gene with longevity [81]. *IL22RA1*, linked to reproductive traits [15], was associated with EBV_udder_ in our study. Longevity was linked to genes from multiple families, like voltage-gated calcium channel *CACNA2D1* [16], the zinc finger genes *ZNF717* and *ZNF613* [20], and the solute carrier gene *SLC2A4RG* [18]. Among these families, we report several novel associations in cattle, like novel links to udder traits were identified for *ZNF277*, *CACNA1G*, *SLC38A7*, and *SLC25A43*. Furthermore, the *DOCK4* gene was between two SNPs associated with EBV_udder_ and EBV_longevity_.

The presence of genes with related functions across the different EBVs suggests that these fundamental biological processes are important for udder health and longevity in cattle. For example, the recurrence of immune response genes for both udder and longevity traits highlights the importance of a robust immune system in maintaining the function. Similarly, the presence of metabolic genes across EBVs underscores the critical role of energy balance and nutrient utilization for milk production and overall lifespan. Efficient metabolism is essential for maintaining cellular function and tissue homeostasis, contributing to both productivity and longevity. Genes involved in cell signaling and transcription regulation indicate the complex genetic control underlying the development, function, and maintenance of these traits. These genes likely orchestrate the cellular processes required for udder development and lactation, and the overall mechanisms determining lifespan.

The proximity of SNPs associated with EBV_udder_ and EBV_longevity_ suggests that the underlying genetic variation might influence both traits. Among the proteins encoded by genes in this region, IFRD1’s involvement in tissue regeneration could also contribute to both udder health and longevity. *BMT2*’s role in metabolic regulation may also be linked to the energy demands of lactation and overall metabolic health, influencing lifespan.

The absence of overlapping SNPs directly associated with multiple EBVs suggests that the primary genetic variants driving each trait might be distinct. However, the presence of genes between nearby SNPs, such as *GPR85*, *BMT2*, *IFRD1*, and *DOCK4*, indicates a genomic region where the genetic influences on udder traits and longevity may be correlated. Both pleiotropy and linkage can lead to significant associations with the studied trait. Here we investigated three traits and no overlapping SNPs were found. In case of EBV udder and longevity, the two SNPs (see Supplementary Table S1, Table 2, and Section 4.3) might be linked to the same causative mutation which has pleiotropic effect, but due to their distance, it seems more plausible that each of them is linked to different causative mutations at different genes, and these -currently unknown- mutations might be under epistatic selection. Fine-mapping studies with more dense marker set could be conducted to pinpoint variants to understand the specific effects of the genes mentioned. The outcome could lead to more precise and effective model to select animals with desirable traits, potentially improving both EBV udder and longevity in parallel of other traits at the same time.

## 5. Conclusions

By exploring the functions of genes associated with EBV_udder_ and EBV_longevity,_ our study revealed a complex genetic landscape with recurring processes. Cell signaling, transcription regulation, immune response, metabolism, and cellular maintenance appear to be important for one or more of these traits. The observation of nearby SNPs associated with EBV_udder_ and EBV_longevity_ highlights a genomic region of particular interest, potentially involving genes such as *GPR85*, *BMT2*, *IFRD1*, and *DOCK4*, which have plausible roles in both traits. Although the genetic drivers of each trait appear distinct, overlapping pathways and closely located variants may underlie correlated improvements in dairy cattle.

## Figures and Tables

**Table 1:** The 62 SNPs associated with the examined traits.

EBV_udder_	EBV_longevity_
34	28

**Table 2:** Functional categories and the genes around the EBV_udder_ and EBV_longevity_ associated with SNPs. Within an EBV category, horizontally, when a gene appears under different functions, the same color was applied for easier visual identification. In the bottom of the table those genes are listed separately, without color coding, which were found between nearby SNPs associated with EBVudder and longevity. For the positions of the markers and the genes see Table S1.

EBV Category	Tissue Structure	Immune Response	Metabolism	Growth, Development, and Differentiation	Tranport and Signaling	Cellular Health and Genomic Integrity	Reproductive Success	Responding to Environmental and Physiological Stress	Mobility and Structural Soundness	Responses to Internal and External Stimuli	Regulation of Gene Expression and Differentiation	Signaling
EBVudder	*CLDN18*, *GRHL3*, *CAPZB*, *SASH1*, *IFRD1*, *EFCAB2*, *DOCK4*	*IFNLR1*, *UBR4*, *NLRC5*	*AKR7A2*, *IFFO2*, *AGK*, *SLC38A7*, *RAB19*	*SOX14*, *SOX5*, *GRHL3*, *IL22RA1*, *EML6*	*MRPS33*, *CNTNAP2*, *CUL1*, *SPTBN1*, *EME1*, *PHB*, *ABCC6*, *UST*, *CACNA1G*					*BMT2*, *GPR85*, *TMEM168*, *LSMEM1*	*IFRD1*, *GPR85*, *ZNF277*, *LSMEM1*	*GPR85*, *BMT2*, *DOCK4*, *TMEM168*
EBVlongevity	*IFRD1*, *EFCAB2*, *DOCK4*	*IL13RA1*, *SELE*	*SELE*, *RAP1GAP2*, *POLR3H*			*PGRMC1*, *SLC25A43*	*SYCP2*, *POLR3H*, *DOCK11*	*SYCP2*, *TRPV3*, *PGRMC1*,	*EFCAB2*, *RANGAP1*, *NGRN*, *NHS*,	*BMT2*, *GPR85*, *TMEM168*, *LSMEM1*	*IFRD1*, *GPR85*, *ZNF277*, *LSMEM1*	*GPR85*, *BMT2*, *DOCK4*, *TMEM168*
Between BTB-01738708 (EBVlongevity) and ARS-BFGL-NGS-111478 (EBVudder)	*IFRD1*, *EFCAB2*, *DOCK4*									*BMT2*, *GPR85*, *TMEM168*, *LSMEM1*	*IFRD1*, *GPR85*, *ZNF277*, *LSMEM1*	*GPR85*, *BMT2*, *DOCK4*, *TMEM168*

## Data Availability

The raw dataset presented in this article is not readily available because the data are part of ongoing studies and are owned by the National Association of Hungarian Holstein Friesian Breeders. Requests to access the datasets should be directed to the second author.

## Supplementary Materials

The following supporting information can be downloaded at: https://www.mdpi.com/article/10.3390/ani16010073/s1, Table S1: The names of the markers associated with udder (U) and longevity (L) EBVs, their genomic positions (B. taurus genome build ARS-UCD1.2), and the closest genes (symbol) to these markers (within 1 million base pairs). Table S2: SNP markers associated with EBVudder (U) and EBVlongevity (L). + sign indicate which row (marker) is associated with the corresponding trait (U or L). The Fst_marker columns are the genetic distance of the markers, Linear Regression −log10(p) columns are the transformed p values calculated via linear regression, Regression Beta is the effect size on a particular trait revealing both the strength and direction of the association between a single-nucleotide polymorphism (SNP) and the phenotype of interest. Negative values are signed by red horizontal bars, positive values are signed by blue and orange bars in case of U and L, respectively. Blue and orange horizontal bars are used in case of Fst, or −log10(p) values of U and L, respectively. Htype −log10(p) columns are the transformed p values calculated via haplotype regression. False Discovery Rate (FDR) values are displayed in case of linear (LinReg FDR) and haplotype regression (HtypeFDR). Table S3: SNP markers associated with EBVudder (U) and EBVlongevity (L). + sign indicate which row (marker) is associated with the corresponding trait (U, L). In each EBV category (U and L) the nucleotide of minor and major alleles are presented together with their regression beta values. Negative values of regression beta are signed by red horizontal bars, positive values are signed by blue and orange bars in case of U and L, respectively. A positive beta means the minor allele, which was the test allele in linear regression, increases the trait’s value; a negative beta means the test allele decreases the trait’s value. Table S4: List of closest genes to SNPs associated with EBVudder, their properties and species investigated in the cited literature. The order of SNPs follows the position they occupy on the chromosome. The referenced literature is under the table. Table S5: List of closest genes to SNPs associated with EBVlongevity, their properties and species investigated in the cited literature. The order of SNPs follows the position they occupy on the chromosome. The referenced literature is under the table. Table S6: List of genes located between nearby SNPs associated with EBVs for udder and longevity, their properties and species investigated in the cited literature. The order of SNPs follows the position they occupy on the chromosome. The referenced literature is under the table.
